# Making Family-Centered Care for Adults in the ICU a Reality

**DOI:** 10.3389/fpsyt.2022.837708

**Published:** 2022-03-24

**Authors:** Ann C. Schwartz, Sarah E. Dunn, Hannah F. M. Simon, Alvaro Velasquez, David Garner, Duc Quang Tran, Nadine J. Kaslow

**Affiliations:** ^1^Department of Psychiatry and Behavioral Sciences, Emory University School of Medicine, Atlanta, GA, United States; ^2^Department of Medicine, Emory University School of Medicine, Atlanta, GA, United States; ^3^Department of Nursing, Grady Health System, Atlanta, GA, United States; ^4^Department of Hematology and Medical Oncology, Emory University School of Medicine, Atlanta, GA, United States

**Keywords:** family-centered, families, patient-centered, intensive care unit (ICU), systemic

## Abstract

Despite the value of family-centered care (FCC) in intensive care units (ICUs), this approach is rarely a reality in this context. This article aims to increase the likelihood that ICU-based care incorporates best practices for FCC. Consistent with this goal, this article begins by overviewing FCC and its merits and challenges in ICUs. It then offers a systemic framework for conceptualizing FCC in this challenging environment, as such a model can help guide the implementation of this invaluable approach. This systemic framework combined with previous guidelines for FCC in the ICU are used to inform the series of recommended best practices for FCC in the ICU that balance the needs and realities of patients, families, and the interprofessional healthcare team. These best practices reflect an integration of the existing literature and previously published guidelines as well as our experiences as healthcare providers, family members, and patients. We encourage healthcare leaders and interprofessional ICU healthcare teams to adopt these best practices and modify them for the specific healthcare needs of the patients they serve and their families.

## Making Family—Centered Care for Adults in the ICU a Reality

Patient-centered care (PCC) is a hallmark of quality care (1). This holistic model emphasizes an empathic, respectful relationship between the healthcare team and patient; ongoing bidirectional communication; and collaborative decision-making regarding care planning that is responsive to the preferences, traditions, and sociocultural backgrounds of patients and family members (2). Unfortunately, given the critically ill status of patients in the intensive care unit (ICU), these individuals often cannot communicate or participate in shared decision-making, the *sine qua non* of PCC. As a result, family members serve as surrogate decision-makers (3). Thus, this interprofessional authorship team that is comprised of physicians from multiple specialties (e.g., psychiatry, pulmonary and critical care, hematology), bedside ICU nurses, and family and clinical psychologists concur with other experts (4) that family-centered care (FCC) is essential and an ethical imperative (5, 6), in the ICU. However, we also recognize this rarely is the reality in this setting.

To help transform this reality, the goal of this paper is to provide healthcare leaders and interprofessional healthcare teams systemically-informed best practices for FCC in the ICU that aim to facilitate family presence, support family members, communicate optimally with families, seek helpful consultations, and optimize operations and the environment. These best practices are the outgrowth of an informal narrative review of the literature on FCC in general and its merits in the ICU, which are summarized briefly at the outset of the paper. They also capitalize on a systemic framework, which is subsequently overviewed. Further, they build upon existing guidelines on FCC in this unique context based on a systematic review (4, 7). Moreover, they reflect the clinical expertise of the interprofessional authorship team comprised of physicians (psychiatry, pulmonary and critical care medicine, hematology, and oncology), psychologists (couple and family, clinical, and clinical health), and bedside nurses. All of the authors are healthcare providers (HCPs) who provide FCC and some also receive ICU care as patients and/or family members.

This focus is relevant for frontline professionals in the ICU such as physicians, advanced practice providers, nurses, social workers, respiratory therapists, etc., who interact with patients and their families on an ongoing basis. It is often nurses and social workers based in the unit that serve many of the critical functions associated with FCC and who can lead the implementation of a FCC culture. It is also relevant to behavioral health professionals, including psychiatrists, psychologists, and advanced practice providers who frequently serve as consultants for ICU patients and often could be and are helpful in meeting the needs of these patients' families. In addition, although often not the case in ICUs with adults, ICUs that serve pediatric populations increasingly are incorporating behavioral health professionals on their integrated care teams in order to ensure that the psychological well-being of these youth and their families is a top priority (8). Ideally, moving forward, family-systems oriented behavioral health professionals can be added to ICU teams caring for adult patients to help ensure that the best biopsychosocial-cultural care is provided for these individuals and their families.

## Family-Centered Care

### Philosophy and Approach

FCC attends to the needs and values of each family unit (4). The patient or their surrogate decision-maker defines the “family,” which may refer to life partners, close blood relatives (children, siblings), extended family, friends, and neighbors. “Family” refers to people who provide support and with whom the patient has a significant relationship.

FCC is guided by five principles (4).

- HCPs and the “family” discuss information and goals openly.
- All perspectives are welcome and cultural, spiritual, and religious beliefs and practices are respected.
- Collaborative decision-making about day-to-day care and life-sustaining treatment is prioritized; all parties have input.
- Negotiation about roles and decisions empowers “families” and capitalizes on their strengths, while ensuring all parties including HCPs are respected.
- With input from families, health systems create and implement family-centered and culturally responsive policies, practices, and systems. These principles focus primarily on the roles and responsibilities of HCPs. They do not guide family members in engaging effectively in this approach to care.

A component of patient- and family-centered care (PFCC), FCC improves patient, family, and HPCs' experience and satisfaction; reduces costs; and bolsters outcomes (7). FCC is associated with lower levels of stress and psychological distress among family members and better interactions with HCPs (9). When HCPs and families partner, FCC is beneficial for HCPs; it enhances their job performance, sense of efficacy, and well-being and lowers their levels of burnout (9). While there are similarities in FCC across all services within a healthcare system, the acuity and high levels of stress associated with the ICU for all parties require unique considerations when delivering this model of care in the ICU.

### FCC in the ICU Setting

#### Benefits

FCC recognizes that family members are critical to their loved one's comfort in the ICU through offering love and companionship, helping with orientation, and responding to questions. Since ICU patients often are intubated or too ill to speak and family members are more knowledgeable about their loved one's wishes than the healthcare team, family members are essential for communicating the patient's thoughts and preferences to the team, advocating on behalf of the patient, and serving as surrogate decision-makers.

Despite the nascent empirical evidence for FCC in the ICU (3, 4), data indicate that FCC maximizes families' ability to be helpful care partners by ensuring they have ongoing contact with their loved one so they can provide them support, information, and meaningful communication (10). It helps families adapt to the ICU and associated unrelenting uncertainty (11) and enables them feel engaged as valued partners. Moreover, FCC fosters collaborative decision-making; it facilitates the family's capacity to make ethical and evidence-informed decisions and ameliorates some of the associated stress (3, 12).

Although interventions that target ICU patients' mental health do not positively impact family well-being (13), strategies relevant to families, such as communicating proactively, providing information, being inclusive, and offering emotional support ameliorate family members' stress levels, “family ICU syndrome,” and “post-intensive care syndrome—family” (14, 15). “Family ICU syndrome” is characterized by physical morbidity secondary to sleep deprivation, psychological distress, cognitive difficulties, and interpersonal conflict (16) and “post-intensive care syndrome-family” refers to high levels of post-traumatic stress, anxiety, depression, and complicated grief reactions after a loved one is discharged from and/or dies in the ICU (17, 18). Further, FCC in the ICU increases families' well-being, engagement, satisfaction, and self-efficacy, as well as decreases lengths of stay and costs (19, 20).

#### Challenges

Yet, there are challenges to the practical implementation of FCC in the ICU. Even when healthcare teams value FCC, they often lack the necessary staff due to a combination of personnel, fiscal, and institutional commitment issues. They also may not have adequate time to invest in FCC; interacting with families and responding to their concerns is time intensive and can take time from other ICU responsibilities, including direct patient care. HCPs frequently express concerns about the time required for family-centered rounds or change of shift reports and the potential negative impact of such discussions on healthcare team members, learners, patients, and/or families.

Some HCPs experience stress when families are the bedside or want to interact with them (4), especially when families have unrealistic expectations about their loved one's care and prognosis or are dissatisfied with the treatment the patient or family receives (21). HCPs also struggle to balance caring for the seriously ill patient with attending to family members' concerns and emotional displays (e.g., yelling during a code while throwing self on the patient to preclude CPR, fainting and diverting attention from the patient) due to staffing limitations, insufficient training, and discomfort with the impact such displays have on patient care. The combination of multiple competing demands and staff shortages often leads to burnout (22), which negatively impacts the emotional well-being of both HCPs and family members. In addition, because of the high acuity, team turnover, and demands of the environment, interprofessional teams often find it difficult to communicate and collaborate (23), which negatively affects families. They also can have difficulties engaging family members in evidence- and value-informed decision-making due to the physical, psychological, and cognitive (e.g., difficulties synthesizing vast amounts of information) challenges of having a family member in the ICU; any history of personal or family conflicts; or previous experiences with healthcare systems (16). Additional, consultations often are not sought at all or in a timely fashion or are unavailable due to resource constraints (24).

Visitation policies, unit rules, limited staff resources, or staff members' attitudes and responses result in many families not feeling welcome (25) or that the care is attuned to their needs or their loved one's best interests (21). This often is the case when families do not experience the healthcare team's communications as transparent, frequent, or responsive to their questions and concerns (18). Communication problems may be most extreme with the when the patient is unable to give permission for the team share information (e.g., intubated, unconscious) and the team determines that sharing information is not in the patient's best interest, which is required by the Health Insurance Portability and Accountability Act (HIPAA). The interpretation of HIPAA is influenced by the extent to which the healthcare team practices FCC. Additional communication challenges that negative impact families relate to HCP's efforts to balance providing information and opportunities for engagement with protecting family members from distress and pain.

Operational and environmental factors serve as organizational barriers to FCC (21, 26). For example, families often are distressed about how the ICU environment itself, such as poor design (e.g., multi-bedded rooms, open ICUs, and insufficient space for family), limited privacy, high noise levels, and lack of resources (e.g., inadequate waiting rooms, limited access to food, and drink) interfere with care that is family-centered (21, 25).

Finally, the COVID-19 pandemic and its associated limitations on visitation made the provision of FCC more challenging and demanding, although it also brought to light how valuable it was for all parties concerned for families to be present or at least engaged in meaningful ways. Moving forward, family members are likely to expect ICU teams to continue to incorporate creative ways to engage loved ones unable to be present as they did throughout the pandemic (5).

## Systemic Framework to Guide FCC in the ICU Setting

Despite the advantages of FCC in the ICU setting and guidelines for its implementation (4, 7), such care is often not a reality. While the aforementioned challenges offer a partial explanation, they do not tell the whole story. Many of these challenges can be moderated by the leadership, infrastructure, processes, and procedures associated with systems-based care.

Systems thinking offers a helpful framework for conceptualizing the multilayered aspects of ICU patients' medical situation, from the biological processes that account for their health status, to the psychological processes that influence their coping and adaption, to the family/social, and cultural contexts in which they are embedded (27, 28). It lays the foundation for viewing families as essential to patients' health and well-being, allies in care, and key members of the care continuum and caregiving team. Systems thinking leads to an understanding of ICU systems as holistic, dynamic, complex, and characterized by reciprocal interrelationships within the system and between subsystems (29, 30).

At a systems level, FCC is most successful in healthcare organizations that prioritize systems thinking and systems-based practice, a core competency in medicine (31). For FCC to become the norm, it must be embraced by organizational leaders and those at the helm of the ICUs. These leaders must emphasize combining quality clinical medicine and physical care; having informative and compassionate interactions with families; creating an inviting and culturally responsive environment; and ensuring that the healthcare team is adequately staffed, resourced, trained, and supported in providing FCC.

For FCC to be truly integrated and advanced within ICUs more universally, healthcare leaders, and professionals must be well-informed about and appreciate the value of a systemic framework. ICU teams that embrace a systemic approach create a culture that emphasizes systemically-informed understandings of patients, families, teams, and health systems and incorporates associated best practices (29). With the support of healthcare leaders, these teams integrate FCC into the infrastructure of the unit such as through the mission and vision, policies and procedures, approach to care, job descriptions, performance evaluations, unit design, documentation, and quality improvement activities (23, 32–34). Unfortunately, many healthcare organization leaders and HCPs fail to adopt a systems approach to thinking, despite how crucial it is to effective system-level redesign. As a result, healthcare systems and leaders often do not buy-into FCC; they fail to support or reinforce HCPs and ICU teams in achieving its aims even though doing so typically is a win-win-win situation (21).

Embracing a systemic approach requires training HCPs to think systemically and appreciate the benefits of FCC based on empirical data and hands-on experience. HCPs are more likely to engage in this approach to care if they receive role modeling, training, and guidance to carry out FCC in the ICU along with the message that this model is valued within the organization and the ICU (21, 22). Ongoing training should inculcate in ICU team members a value on viewing families as care team members and partners who can be a resource and support, rather than as visitors or intruders who cause them undue burden. To assist them in carrying out this value, such training must be designed to teach HCPs how to individualize care to each family, integrate family members as desired in the patient's care, and harness the family's strengths in support of the patient's care. This training should also teach HCPs the skills necessary for incorporating family members' expertise in patient and family values and needs into the biopsychosocial-cultural care that is provided (5). It must help them become more facile at making decisions that include multiple points of view in a manner that attends to the nested and interacting levels of the healthcare system (e.g., patient, family, team, ICU, hospital, political, and economic context).

ICUs that adopt a systemic framework also must invest in making unit-based changes to improve care delivery to patients and families. One beneficial change for units to consider is adding a family navigator and/or family support specialist to the interprofessional care team who can serve as a bridge between the healthcare team and the family. Such an individual may be a family-systems trained behavioral health professional such as family psychologist, psychiatrist, medical family therapist, or advanced practice professional. This individual may assume primary responsibility for educating each family about FCC in the ICU. This involves conveying that FCC is a dynamic relationship between families and the team, gathering information about family members' expectations, and providing information about reasonable expectations for FCC and the patient's likely course. It also involves offering tools for the family to participate in FCC (e.g., teach them about procedures and basic care) and acknowledging and normalizing the family's understandable range of emotions (e.g., shock, fear, and anger) and trauma. Moreover, this individual can serve as a critical function in providing family members with practical and emotional support and fostering discourse between the healthcare team and the family. This team member should be empowered to facilitate and mediate team-patient-family communication, support shared decision-making, help the family navigate differences, and attend to the emotional well-being of all parties (34, 35). There is evidence incorporating a systems-thinker and practitioner on the team improves satisfaction with care for HCPs and families alike (35). When such a designated professional is not available, the functions they serve must be assumed by other team members.

Another approach to improving FCC is to create and utilize an advisory group comprised of prior ICU patients and family members (36). Ideally, their input is sought on ways to make ICU operations and environment more patient-and family-centered. They also can be engaged in problem-solving solutions for navigating the challenges of balancing staff responsibilities and demands with the needs of patients and their families.

## Best Practices for FCC in the ICU

The five best practices build upon evidence-informed guidelines for FCC in ICUs with adult patients (4, 7). They expand upon these guidelines in three ways. First, they are guided by a systemic framework. Second, they incorporate recent evidence and the collective wisdom and clinical experience of the interprofessional authorship team. Third, the best practices are delineated in a comprehensive fashion and include specific implementation strategies. We believe that ICUs that employ these best practices will more effectively engage and support family members as respected collaborators in care, foster families' understanding of the situation and their new roles, improve healthcare team-family communication, and enhance family participation in decision-making (37). While no ICU can incorporate all practices and must decide which to prioritize in their policies, procedures, and processes based on their setting and values (3), systemically informed FCC must be a core value.

### Encourage and Facilitate Family Presence

Families play critical roles in caring for their loved ones in the ICU; they partner with HCPs in providing care, aid in decision-making, and improve safety and quality. Twenty-four hour visitation and ongoing access to information and opportunities for hands-on-care and support are associated with positive family outcomes and satisfaction (38). Thus, as detailed in previous guidelines, policies related to family presence should be open, flexible, and unlimited (4, 7) and optimally include open-door visitation (22, 38, 39), with restrictions only when necessary. Visitor policies need to be followed consistently and not used to control the unit and/or particular families or family members.

Such policies are best implemented if HCPs are informed about the benefits of enhanced visitation and embedded in a unit culture that values families' preferences about their presence and their engagement. This can be accomplished by HCPs educating families about how being at the bedside may support or stress the patient and ways to respond accordingly. It requires healthcare team—family collaboration in determining when the family should be present (e.g., patient becomes calmer or better oriented, family feels too stressed when not present) and when to leave (e.g., increased agitation in patient, family member needs sleep). HCPs should promote helpful contact by guiding family members in caring for and supporting the patient (e.g., feeding, facilitating range of motion exercises, bathing, and reading to them) or personalizing the patient's room so it is familiar and conveys who they are as a person (39). HCPs must learn from the family about the patient's likes/dislikes to inform future interactions (40).

Prior guidelines recommend offering family members the option to witness procedures or medical interventions (e.g., cardiopulmonary resuscitation) (CPR) (4, 7) given evidence that many relatives desire to be present and find such presence beneficial and that family presence does not disrupt patient care (41). Families who opt to be present should be provided with support and guidance from a designated staff member so they are not unduly traumatized (22, 41). The same should occur if family members are not physically present but desire such information.

Encouraging presence also means including family in staff communications about the patient (4). Families appreciate such inclusion and it positively impacts the patient and family experience (42). Families can be included if nursing change of shift reports and interprofessional rounds occur at bedside and are family-centered. Family members should be informed about the timing and purpose of these activities, appropriate times to ask questions (e.g., during and/or outside of rounds) and realistic to expect responses and updates, and the reason these activities cannot be at bedside (e.g., patient in isolation limiting number of people in the room). HCPs are most open to bedside processes if they know they reduce errors in information transfer, foster collaboration and dialogue, and increase family satisfaction (42, 43).

It is important for interprofessional [e.g., ICU physician(s), nurses, social workers, behavioral health professional(s), family navigator or support person, other team members] family-oriented care conferences to be held (15, 22). Ideally, these conferences cover introductions, goals of the conference, patient's medical situation and prognosis, and potential future decisions and outcomes. Often these conferences need to involve end of life conversations that attend to the family's definition of quality of life, patient's view if possible, and patient and family values. The family should be invited to ask questions that are responded to and their feelings and perspectives should be acknowledged. Family members should be engaged in shared decision-making including about complex issues. These conferences should conclude with a summary of goals, decisions, and next steps.

When family members are unable to be physically present, the healthcare team should engage them virtually (daily if possible) so they can offer their loved one support and comfort. This can be facilitated by having a telephone in every room, making Ipads available, and/or using the patient's personal device. Units need to incorporate technology (e.g., smartphone apps, social media) that enable family members to carry out critical functions for the patient, regardless of whether they are at the bedside. While the COVID-19 pandemic has made FCC in the ICU more challenging, it has advanced our capacity to communicate effectively with families *via* technology when they are unable to be at the bedside. These advancements must continue to be integrated into ICU care in the future.

### Support Each Family and Its Members

Existing guidelines emphasize supporting families (4, 7) so they feel less overwhelmed, distressed, and traumatized (18). This involves prioritizing friendly and compassionate interactions with family members (16). Examples include HCPs introducing themselves and their role on the team to the family repeatedly, orienting families to the setting, being mindful not to ask questions that are perceived as repetitive and/or unnecessary, and explaining their actions as they perform them. All such interactions must reflect empathy and kindness as well as competence.

In accord with medical family therapy, which builds upon a systemic framework, supportive interventions should aim to bolster family members' agency and personal choice, foster their interpersonal connections, and promote family functioning and well-being (44, 45). This entails attending to family members' psychological reactions, needs, and wishes; identifying their strengths; helping them manage their lives and stress; encouraging them to prioritize self-care and accessing resources; and providing them necessary resources (e.g., lip balm, exercise room in the hospital, library services, and internet access).

Prior guidelines recommended specific mechanisms for supporting families. The first is education, which involves providing basic information that fosters family members' comfort in the ICU. It entails conducting a family meeting that focuses on information about the ICU, ICU rules and their rationale, machines in the patient's room, realistic expectations, and roles they may play (e.g., companion, assistant, representative, and planner). During these discussions, families should be prepared for potential setbacks and negative outcomes while also being given appropriate hope. While optimally such a meeting and associated support is provided from the outset of the admission (i.e., within the first 24–36 h), family members may not be present or reachable initially, and thus may need to occur at a later time. Unfortunately, a standard orientation process can be challenging because of variability in families' expectations about ICU care, their stress levels, and their capacity to cope. But it must be standard practice to transmit relevant education and information to family members in a timely fashion. Families also can benefit from receiving written materials (e.g., brochures, booklets) and/or having videos that review the aforementioned information and address pertinent topics (e.g., death and dying, grief).

A second mechanism is ICU diaries, which are documents crafted daily by family and/or staff (19, 46). Both family members and HCPs should be encouraged to chronicle the events leading to an ICU admission or intubation and subsequent progress or setbacks and express related emotions. HCPs should commit to family that if they leave the bedside, a HCP will document in the diary and contact the designated family member if there is a noteworthy change. These ICU diaries should be shared with the patient during their recovery or upon discharge so they learn what they experienced or with the family if their loved one dies to assist them with debriefing or reminiscing.

### Prioritize Communication With Families

Existing guidelines highlight the value of ICU team—family communication that is respectful, emotionally attuned, empathic, supportive, and patient-focused as well as family-centered (4, 7). For this to be realized, it often requires creating a plan in which a point person for the healthcare team is assigned (individual or role) to communicate regularly with the designated family member(s) and this person's word should be considered official when there are mixed messages. The family needs to be informed how to contact this individual if they have questions or concerns and if they are unavailable, who they should reach out to and how. Similarly, there needs to be a clear understanding among all parties about the family member who will serve as the liaison for information, questions, and concerns between the healthcare team and the family. If the patient has designated a decision-maker, this person is easily identified. If not, the healthcare team should ask the family to designate one or two point people. In accord with the growing OpenNotes movement, these designated family members may meet the requirements for being proxies (i.e., care partners) who can access the patient's electronic health record and review medical information in that way.

Intentional family-centered communication involves healthcare team members sharing information regularly (i.e., at minimum daily) and in an honest, transparent, timely, and proactive manner; ensuring it is understandable and realistic; and not glossing over bad news (25, 39). When possible, they can provide information visually (e.g., radiographic imaging). HCPs need to repeat critical information as often as necessary and with patience. Further, it helps if they acknowledge the challenges providing FCC and ask each family for guidance on doing so optimally for them. HCPs must do their best to mitigate problematic interactions through discussion and shared problem-solving, rather than avoidance or hostile communications.

Family-centered communication requires HCPs to listen actively to family members; attend to their values, feelings, concerns, questions, and goals; and mobilize their resilience and enhance their psychological well-being as they function as care team members and surrogate decision-makers (44). A structured approach to such communication often is valuable. One helpful mnemonic is “VALUE” (47):

- V = value family statements,
- A = acknowledge family emotions,
- L = listen actively to the family,
- U = understand the patient as a person, and
- E = elicit family questions.

The use of a structured communication tool results in greater satisfaction and more realistic expectations about survival (48).

Related to ensuring effective communication, prior guidelines also emphasize shared decision-making, which enhances family satisfaction with care and clinicians' sense of efficacy (4, 7). The following are specific strategies for engaging families in shared decision-making (12, 16, 49–51). First, HCPs need to solicit family members' wishes about their preferred level of involvement and gather information about the family's goals of care and perceptions of the patient's priorities related to treatment planning. They then need to identify clear decision points, provide pertinent information about the patient's current clinical situation and potential options, elicit family members' perspectives and help them navigate differences in these points of view, guide the family toward a final decision that hopefully has buy-in from all parties, and assess the family's comfort with the decision(s). This process, which can be repeated whenever there is a decision to be made, can be facilitated by decision aids. Collaborative decision-making in which the family is empowered to serve as a true partner with the healthcare team takes time; involves a focus on medical and nonmedical goals; and requires HCPs to listen to, respect, and accept the input (49, 50). The process of collaborative decision-making should be documented in the electronic health record (52).

Critical to effective communication is HCPs overcoming their reluctance to having difficult conversations, such as about end-of-life (49). When such conversations are held, team members known well to the patient or family should be present. The discussions should occur early and often enough in the trajectory of care that patient and family input truly matters (40, 49).

### Seek Consultation

An additional guideline pertains to accessing one or more consultation services in support of FCC (4). Such consultations often reduce family members' levels of psychological distress and increase their satisfaction with care, increase the attainment of clinical consensus, and shorten patients' lengths of stay (18, 43). Seeking appropriate consultations aligns with a systemic framework's emphasis on interrelations between systems, such as units within the healthcare system.

The following are three examples of systemically-informed consultations. For many patients in the ICU, palliative care consultations should be episodic or ongoing (53). These consultations, which should occur in collaboration with the ICU team as discussed above, typically need to focus on goals of care and end-of-life decision making. Palliative care consultants often can assist ICU teams in communicating the prognosis of seriously or terminally ill adults to families and patients, respecting family members' needs and autonomy (if patient is not competent to make decisions) about life sustaining treatment vs. end of life care, and attending to differences within families when these emerge. These consultants often are excellent models for ways ICU teams can be attuned to and respectful of family members' cultural, religious, and spiritual beliefs related to end-of-life care, as well as their associated emotional and spiritual needs. As a second example, ethics consultations can support patients, families, and the team in ethically challenging situations (54). Such consultations can be useful for clarifying goals of care and addressing disagreements between the ICU team and surrogate decision-makers. A third example is consulting with spiritual support/pastoral care if consistent with the family's wishes (53). Members of the spiritual support/pastoral care team can serve an invaluable function in listening to family members' emotional pain, supporting them in grappling with existential questions, offering compassion, and providing spiritual/religious support. The inclusion of a chaplain in ethics discussions can assist family members in determining the extent to which decisions are consistent with patient/family beliefs and providing them support for the decisions made. It behooves HCPs to recognize the critical role chaplains play in advocating on behalf of patients and families and serving as ambassadors between the healthcare team and the patient-family system.

### Optimize Operations and the Environment

Previously detailed guidelines related to optimizing operations and the environment (4, 7) are in keeping with a broad conceptualization of systems that recognizes that human behavior occurs within a contextual matrix of individual, interpersonal, environmental, or macrosystemic factors (30, 55). These environmental and macrosystemic factors must be considered to ensure FCC. In other words, hospitals need policies, procedures, and processes that promote FCC in the ICU in concrete ways. In keeping with the movement toward humanizing care within ICUs, these policies, procedures, and processes need to support open visitation and family engagement, foster positive communication (e.g., among HCPs; team-patient-family), incorporate mechanisms to trigger early conversations about goals of care, and ensure humane operations and environment (39). They need to lay the foundation for HCPs to be intentional about supporting families and communicating with them effectively, mitigate against and ameliorate family ICU syndrome and post-intensive care syndrome—family, and prioritize compassionate end-of-life care. In a related vein, healthcare organizations need to create systems that support FCC (49). Examples of this include making available relevant technological supports and developing a section of the electronic health record in which HCPs record information shared with the family, goals of care conversations, family input in and conflicts about decisions. Such systems need to embrace quality improvement efforts designed to monitor and assess indicators of FCC, evaluate family satisfaction with ICU care, and examine HCP's perceptions of support and necessary resources for such care. Moreover, healthcare systems overall and ICUs specifically must hire and retain staff that prioritize FCC and train staff to be competent in this approach. Staffing models need to be refined and optimized to include people with expertise in FCC who can meet the unpredictable workloads and demands of FCC.

Units must strive to create a welcoming environment in which family members feel respected as valued members of the care team (25, 39). The physical nature of these environments should be family-friendly, with adequate places to sit, sleep, and take a break. HCPs should facilitate nighttime rest by minimizing noise levels and lights and ensure families have access to nourishment when desired. Healthcare systems need to devote resources for family self-care (e.g., bathrooms and showers, kitchens, and laundry rooms) and make accessible spaces where family members can find serenity (e.g., gardens, Zen rooms).

Finally, ICUs must care for their HCPs (39) by creating organizational conditions and environments that support interprofessional teamwork, emphasize competency attainment associated with teamwork (e.g., coordination, communication, and adaptability) and interprofessional collaboration, and promote mechanisms for accurate transfer of patient information among team members. Interprofessional teamwork improves patient outcomes, team functioning, patient and family satisfaction, and provider well-being (23, 56, 57). ICUs that care about their HCPs prevent and address burnout through educating people about this syndrome, encouraging the use of strategies to bolster their resilience, and transform the organizational culture from one that engenders burnout to one that supports HCPs well-being.

## Concluding Comments

Moving forward, for FCC to become the *sine quo non* of quality care, studies on its implementation, added benefits, and outcomes in the ICU are critical. Such investigations may examine key elements of FCC in this setting, ways to tailor care to each family and unit, and strategies for incorporating FCC into daily practices. They may focus on developing, executing, and evaluating new approaches to improve families' well-being and quality of life and innovative programs to guide families in participating in FCC (58). The benefits of a systemically informed behavioral health professional on the team for patient, family, and staff well-being and outcomes should be examined. Studies must address ethical challenges, such as family engagement in care planning and delivery in light of legislation (e.g., HIPAA) (59). Such research will be most valuable if family members and former patients are partners on research teams and inform the questions being addressed, the constructs assessed, and the interpretation of the findings.

There are genuine challenges to implementing FCC in ICUs that serve adults. Embracing this model of care requires healthcare systems and ICU teams to make tradeoffs, some of which are quite challenging. It is possible that some of these compromises could lead to negative consequences and even harm. Thus, quality improvement initiatives must ascertain the advantages and disadvantages of shifting an ICU culture toward one that is family-centered; guide efforts to mitigate negative outcomes; and inform decision-making when the selection of FCC processes or procedures has a problematic impact on patients, families, and/or units.

Further development, implementation, modification, and dissemination of FCC programs in ICUs with adult patients requires input from all parties. This will help ensure that care both responds to the preferences, needs, and values of patients and families and respects the practical and emotional demands such care places on HCPs.

In closing, despite its challenges, FCC in the ICU promotes the health and well-being of patients, family members, and HCPs. By working as partners, all parties are empowered to collaborate as allies in the patient's healthcare journey.

## Author Contributions

AS and NK assumed primary responsibility for writing the manuscript, although all other authors participated actively in editing the manuscript. SD offered expertise as a patient, as well as a patient- and family-centered care psychologist. HS offered her views based on providing family-centered psychological services in the ICU. AV provided expertise as an ICU physician and leader. DG offered expertise as an ICU nurse. DT provided expertise as a specialist who consults frequently in the ICU. All authors participated in the conceptualization of the manuscript and the determination of the recommendations. All authors contributed to the article and approved the submitted version.

## Conflict of Interest

The authors declare that the research was conducted in the absence of any commercial or financial relationships that could be construed as a potential conflict of interest.

## Publisher's Note

All claims expressed in this article are solely those of the authors and do not necessarily represent those of their affiliated organizations, or those of the publisher, the editors and the reviewers. Any product that may be evaluated in this article, or claim that may be made by its manufacturer, is not guaranteed or endorsed by the publisher.
